# PseudoSorter: A self-supervised spike sorting approach applied to reveal Tau-induced reductions in neuronal activity

**DOI:** 10.1126/sciadv.adr4155

**Published:** 2025-03-14

**Authors:** Marius Brockhoff, Jakob Träuble, Sagnik Middya, Tanja Fuchsberger, Ana Fernandez-Villegas, Amberley Stephens, Miranda Robbins, Wenyue Dai, Belquis Haider, Sulay Vora, Nino F. Läubli, Clemens F. Kaminski, George G. Malliaras, Ole Paulsen, Gabriele S. Kaminski Schierle

**Affiliations:** ^1^ Department of Chemical Engineering and Biotechnology, University of Cambridge, Cambridge, UK.; ^2^ Electrical Engineering Division, Department of Engineering, University of Cambridge, Cambridge, UK.; ^3^ Department of Physiology, Development and Neuroscience, University of Cambridge, Cambridge, UK.

## Abstract

Microelectrode arrays (MEAs) permit recordings with high electrode counts, thus generating complex datasets that would benefit from precise neuronal spike sorting for meaningful data extraction. Nevertheless, conventional spike sorting methods face limitations in recognizing diverse spike shapes. Here, we introduce PseudoSorter, which uses self-supervised learning techniques, a density-based pseudolabeling strategy, and an iterative fine-tuning process to enhance spike sorting accuracy. Through benchmarking, we demonstrate the superior performance of PseudoSorter compared to other spike sorting algorithms before applying PseudoSorter on MEA recordings from hippocampal neurons exposed to subneuronal concentrations of monomeric Tau as a model for Alzheimer’s disease. Our results unveil that Tau diminishes the firing rate of a subset of neurons, which complement our findings observed using conventional electrophysiology analysis, and demonstrate that PseudoSorter’s high accuracy and throughput make it a valuable tool for studying neurodegenerative diseases, enhancing our understanding of their underlying mechanisms, as well as for therapeutic drug screening.

## Teaser

Advanced spike sorting based on self-supervised learning unveils the effects of Tau on hippocampal neuronal activity.

## INTRODUCTION

Microelectrode arrays (MEAs) have revolutionized the landscape of neuroscience research by enabling prolonged and extensive monitoring of local field potentials from neurons over a large area. The technology enables noninvasive recordings of diverse spatial and temporal neuronal signals, thus providing additional information as compared to other electrophysiological techniques such as patch clamping (*1*). Despite these advantages, MEAs encounter challenges in both fundamental and therapeutic neuroscience research (*2*, *3*). These challenges arise from the inherent complexity of neuronal signal analysis, given that each electrode records signals from multiple neurons and various noise sources (*2*, *4*–*6*), resulting in complex datasets.

A critical bottleneck in the analysis of MEA-recorded data is spike sorting, the process of attributing recorded spike signals, i.e., extracellular action potentials, to individual source neurons. This step is pivotal for enhancing our understanding of neuronal function and dysfunction (*5*, *7*, *8*), as spike sorting has been used to study how neurons with distinct extracellular spike waveforms exhibit unique responses to experimental treatments or pathological conditions. For instance, previous studies have demonstrated that spike waveform features can distinguish different neuronal subtypes (*9*) and have been applied to investigate the effects of pharmacological agents or neurological disorders on neuronal populations (*10*, *11*). However, despite the existence of a variety of spike sorting methodologies (*12*–*20*), limitations in accuracy, reliability, scalability, and reproducibility (*7*, *21*, *22*) remain. Accurately identifying the number of neurons present and distinguishing their spikes becomes particularly challenging in densely packed neuronal cultures or brain tissue, where signal overlap is common or when neurons fire so rarely that they are not detected among more abundant signals (*5*, *23*). Moreover, the intrinsic variability in spike shapes, electrode drift or damage, and the presence of noise further complicate accurate assignments (*13*, *22*). Hence, addressing these challenges is crucial for advancing the capabilities of MEAs and unlocking their full potential to unravel the intricacies of neuronal activity.

Machine learning (ML) approaches, with their capacity to handle large datasets and learn complex patterns, are well suited to address the complexity of spike sorting. Traditional methods, such as those based on principal components analysis (PCA) (*19*, *24*, *25*), build a strong foundation for identifying neuronal activity but often face limitations in scalability, noise tolerance, and handling overlapping spikes. In contrast, recent advancements in data-driven ML approaches to spike sorting (*8*, *22*, *26*–*37*) have demonstrated improved accuracy and fast online processing. Building on these developments, we present PseudoSorter, an ML approach to spike sorting that incorporates self-supervised learning, data augmentation, and a pivotal density-based pseudolabeling strategy. Benchmarking on large-scale simulated datasets (*37*, *38*) reveals that PseudoSorter outperforms existing algorithms.

We next evaluate the capabilities of PseudoSorter using an in vitro neuronal culture model of neurodegeneration. In this model, primary hippocampal neurons are exposed to monomeric Tau, a protein intricately linked to Alzheimer’s disease (AD), as previously established by Mandelkow and Mandelkow (*39*). Our prior work demonstrates that extracellular uptake of monomeric Tau alone is sufficient to induce Tau pathology (*40*) and that Tau transfers between cells in a prion-like manner (*41*, *42*). This phenomenon underscores the significance of Tau, conventionally an intracellular protein, gaining access to the extracellular milieu and being reinternalized by neighboring neurons. These cellular dynamics are intricately linked to the progression of AD pathology, highlighting the relevance of our investigation.

Hence, the pivotal question is whether such extracellular monomeric Tau is also capable of inducing neuronal signaling defects in primary hippocampal neuronal networks. This has prompted us to expose the neurons to subneuronal concentrations of monomeric Tau, i.e., concentrations that are below the physiological concentration of Tau in neurons (~2 μM) (*43*, *44*), and evaluate the outcomes using either patch-clamp experiments or MEAs plus/minus PseudoSorter*.*

By investigating the effect of extracellular Tau on excitatory synaptic transmission using either whole-cell voltage-clamp or MEA recordings, we find that the addition of 1 μM Tau to hippocampal neurons prevents a stimulation-induced enhancement of excitatory synaptic transmission and that the overall spike rate of neurons is reduced, respectively. We then use PseudoSorter to separate between neurons with different spike shapes and reveal that PseudoSorter is able to detect neuronal clusters that experience a loss of firing capacity following Tau treatment alone.

These results are of significance not only to the field of AD but also to the study of traumatic brain injuries, both of which are strongly related to Tau pathology, as we show how subneuronal concentrations of monomeric Tau, once present in the extracellular space, can strongly impair neuronal signaling. PseudoSorter, because of its capacity to specifically classify individual neuronal spikes, is able to pick up the small changes that occur in a population of hippocampal neurons and thus permits us to study Tau-related pathology with much higher precision than using traditional MEA analysis approaches. In addition, PseudoSorter, owing to its high accuracy, can also be applied for fundamental inquiries into neural network dynamics as well as for the exploration of brain-computer interfaces. PseudoSorter is freely available, offers a high level of adaptability, and uses an agnostic approach to the experimental settings, making it broadly applicable.

## RESULTS

### PseudoSorter: Self-supervised density-based pseudolabeling for spike sorting

**Fig. 1.**
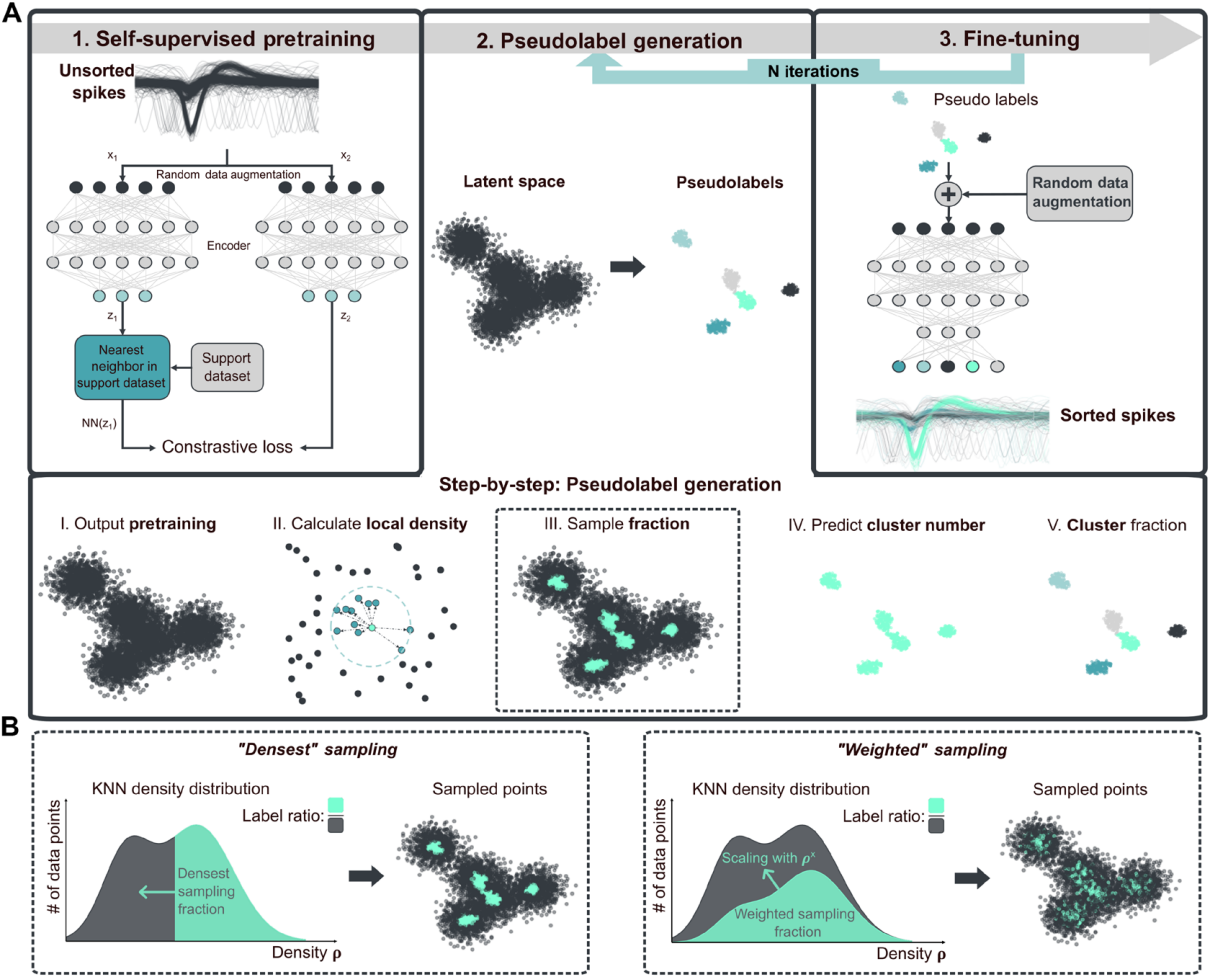
Schematic workflow of PseudoSorter. (**A**) Top row: Three-step workflow of PseudoSorter. Self-supervised pretraining (*45*) on unsorted spike shape recordings yields an encoding model that produces a representative latent space. On the basis of the latent space, an iterative process of pseudolabel generation and fine-tuning is executed, ultimately leading to high-accuracy classification for each spike shape recording. In the fine-tuning step, the encoding model is trained on the previously generated pseudolabels (semisupervised problem) in a classification model. Lower row: Detailed five-step workflow to generate pseudolabels from latent space. Pseudolabels are sampled from whole latent space based on the local KNN density of each sample. The number of present clusters is predicted via the elbow method, and K-means++ is used to allocate all sampled points to one of the pseudoclasses. (**B**) Two strategies to sample high-confidence samples from a dataset based on local KNN density. “Densest” describes the naïve approach of sampling the densest fraction of the dataset. Alternatively, the “weighted” sampling strategy that inherently favors points of higher local density but still captures a representative fraction of the dataset is proposed.

PseudoSorter encompasses three main steps (Fig. 1A). First, a self-supervised encoding model is pretrained to learn invariant features from spike waveforms. Next, pseudolabels—representing high-confidence estimates of neuron classifications—are generated by sampling a fraction of the dataset with high local density, capturing representative features from both common and rare spike patterns. Last, these pseudolabeled samples are used to fine-tune the model, progressively refining its accuracy through iterative updates.

### Pretraining

In the pretraining phase, we use a self-supervised contrastive learning framework, specifically adapted from the Nearest-Neighbor Contrastive Learning of Visual Representations (*45*) algorithm for spike waveform data. The input consists of preprocessed, unclassified spike waveforms. These waveforms are augmented using Gaussian noise with a maximum relative noise level of 0.075, acting as the contrastive augmenter to introduce stochastic variations that mimic realistic alterations in spike shapes. The augmented waveforms are passed through a fully connected encoder network with a latent bottleneck of 10 dimensions. The encoder architecture comprises dense layers with ReLU activations, following the dimensions [63, 500, 500, 2000, 10]. The projection width is set to 10. The primary objective here is for the model to learn invariant features by contrasting each spike waveform against its nearest positive neighbor in a dynamically updated memory queue. Positive samples are defined as augmented versions of the same spike waveform, created through stochastic augmentations, here using Gaussian noise, that preserve the core features of the waveform. The contrastive loss is formulated as

$${ℒ}_{\text{contrastive}}=-\text{log}\frac{\text{exp}\left[\text{sim}\left(z_{i},z_{i}^{+}\right)/\tau \right]}{\sum_{j=1}^{N}\text{exp}\left[\text{sim}\left(z_{i},z_{j}\right)/\tau \right]}$$

where *z*_*i*_ and *z*_*i*_^**+**^ are the embeddings of the anchor and positive samples, τ is the temperature parameter set to 0.1, and sim(·) denotes the cosine similarity. This formulation aims to maximize the similarity between augmented views of the same spike waveform while minimizing it against other samples in the dataset. The pretraining is conducted over 25 epochs with a batch size of 256, using the Adam optimizer with a learning rate of 1 × 10^−3^.

### Pseudolabel generation

Following pretraining, the spikes are represented in a latent space where similar spikes are expected to cluster together. For the generation of pseudolabels, a subset of points believed to have well-defined embeddings within the latent space is sampled—these representative points serve as a basis to fine-tune the encoding. Instead of clustering the full dataset at once, the aim is to identify a subset of the data that are inherently easier to classify.

The generation of pseudolabels involves several substeps (Fig. 1A, lower row). First, the local density of the latent space is calculated via the inverse of the average distance to K nearest neighbors (KNN density), setting K to 0.5% of the dataset size. Given a sampling fraction of the pseudolabels, two different sampling methods can be used (Fig. 1B). “Densest” sampling describes the naïve approach of sampling the densest fraction of the dataset. This strategy has the disadvantage that the densest fraction of the dataset is less likely to represent the full dataset and, therefore, might generalize poorly in the fine-tuning step. Instead, a “weighted” sampling method is proposed. Here, an exponential decay is overlaid with the normed density distribution. This leads to a high relative sampling chance for high-density points, while maintaining a nonzero sampling chance for even the least dense points. Biasing toward high-density points, i.e., high confidence as assumed in the center of the clusters, ensures that the subsequent pseudolabels reflect the intrinsic distribution of the data, encompassing both high-density regions corresponding to frequent spike types and lower-density areas where rarer or unique spike patterns may reside. Using K-means++ (*46*) clustering on the sampled points, pseudolabels are assigned, i.e., a preliminary neuron class is assigned to each point within the sub–latent space. These pseudolabels serve as proxies for the true classes, effectively transforming the unsupervised learning problem into a semisupervised one. As K-means++ necessitates the number of distinct clusters, the elbow method (*47*, *48*), which identifies the optimal number of clusters by locating the point where the decrease in within-cluster variance slows substantially (forming an “elbow” on a plotted graph), is used to estimate the number of source neurons on 50% of the dataset, selected by densest sampling.

**Fig. 2.**
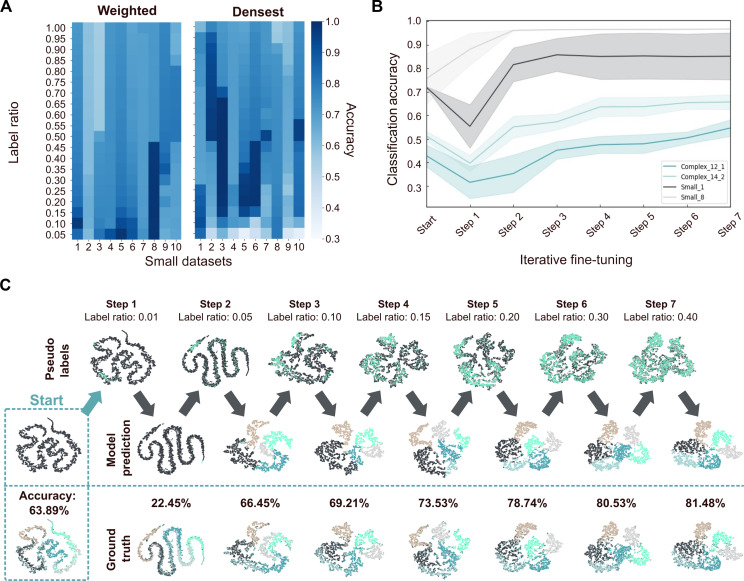
Pseudolabel generation and fine-tuning iteratively improve spike sorting accuracy. (**A**) Heatmap showing the classification accuracy of sampled points for pseudolabel generation for the Small datasets of “weighted” (left) and “densest” (right) sampling method. The *y* axis describes the fraction of the dataset that is sampled, and accuracy describes the measured K-means++ classification accuracy achieved on the sampled data for each sampling method. (**B**) Measured accuracy during iterative pseudolabel generation and fine-tuning for different example datasets (Complex_12_1, Complex_14_2, Small_1, and Small_8) are plotted. Shown are mean (dark lines) and SD (shaded area) for five repeated runs per dataset. (**C**) Illustrations of the iterative fine-tuning process with seven steps showing incremental improvements in model accuracy and the respective two-dimensional t-distributed stochastic neighbor embedding (tSNE) (*70*) visualization of the latent space. The unlabeled latent space produced by pretraining is used as the starting point (Start). The top row indicates the sampled subset of the dataset of pseudolabeling, the middle row shows the predicted classes of the model at each step, and the lower row shows the corresponding ground-truth classes. The initial accuracy and stepwise accuracies are given for each step. Dataset: Complex_6_3.

Sampling a certain fraction of the dataset for high-confidence pseudolabels poses the challenge of identifying a fraction that works well for every dataset. It is demonstrated that choosing one fixed fractional value is not a feasible approach as different datasets exhibit different fractional pseudolabel qualities (Fig. 2A) for either sampling method. To solve this problem, an iterative process is introduced, alternating between the steps of pseudolabel generation and fine-tuning with an increasing fraction of sampled pseudolabels. As shown in Fig. 2B, this iterative approach achieves high-accuracy results for diverse datasets. Even if high accuracy is already achieved early (Fig. 2B, Small_1 and Small_8), this accuracy can be maintained and is not lost with further fine-tuning. At the same time, other datasets require the full range of steps and incrementally increase the overall performance over time (Fig. 2B, Complex_12_1 and Complex_14_2). In this way, improved accuracy compared to choosing a fixed fractional value is achieved while reducing the required parameter choice.

### Fine-tuning

For the fine-tuning stage, the pretrained encoder is appended with a final classification layer, representing the putative neuron classes. We iteratively update the model using an expanding set of pseudolabels, starting with a fraction of 1% and increasing up to 40% of the dataset (fractions: 0.01, 0.05, 0.10, 0.15, 0.20, 0.30, and 0.40). Data augmentation using Gaussian noise with a maximum relative noise level of 0.1 is applied during fine-tuning to enhance generalization and to mitigate overfitting. Each iteration involves 50 epochs with a batch size of 128. After each iteration, the latent space embeddings are recalculated, and different pseudolabels are sampled, progressively refining the model’s classification accuracy. This process is illustrated for an example dataset (Complex_6_3) in Fig. 2C. Details of key hyperparameters and training settings are provided in table S1. All models were trained on an NVIDIA A-100-SXM-80GB GPU.

### PseudoSorter benchmarking study on simulated data shows improved spike sorting performance

**Fig. 3.**
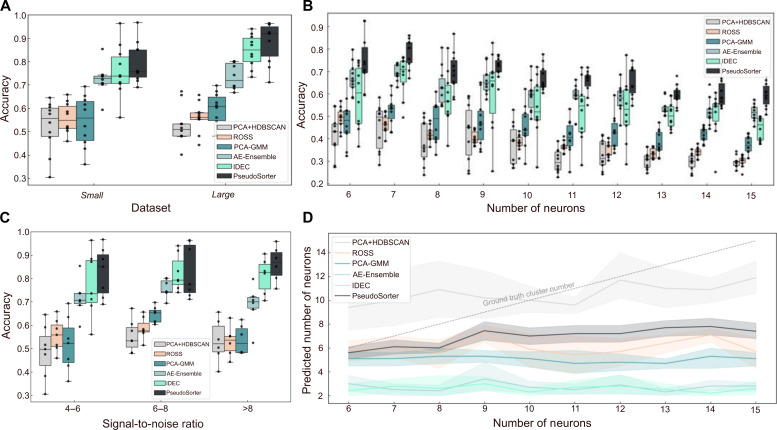
Evaluation of spike sorting accuracy and neuron number prediction across diverse data size and complexity levels illustrates PseudoSorter’s superior performance. (**A**) Boxplots depicting the accuracy of spike sorting for Small and Large datasets (*N* = 20 datasets, 10 datasets each), comparing PseudoSorter with the PCA + HDBSCAN (*19*), PCA-GMM (*24*, *25*), AE-Ensemble (*31*), ROSS (*33*), and IDEC (*37*, *49*) methods. (**B**) Boxplots showing accuracy as a function of increasing number of neurons (Complex datasets, *N* = 100 datasets, 10 per number of neurons), maintaining constant dataset size. (**C**) Boxplots representing the accuracy of each spike sorter for different regimes of signal-to-noise ratios (*N* = 20). (**D**) Trend lines with shaded interquartile range (IQR) areas illustrating the median predicted neuron numbers for all Complex datasets (*N* = 100) with the respective increasing number of neurons.

To quantify PseudoSorter’s performance compared to existing alternatives, single-channel extracellular recordings have been simulated with NeuroCube (*38*) for an extensive benchmarking study. The datasets have been designed to roughly match the expected quality and noise levels of in vitro experiments (see the “Benchmarking datasets” section). We first investigate how scaling the amount of neural recording data, i.e., the number of spike shape recordings, by one order of magnitude, while maintaining the same complexity—in this case, featuring spikes from five neurons—affects spike sorting performance (Fig. 3A). Shown are the classification accuracies across Small (~100,000 spikes per dataset) and Large (~1,100,000 spikes per dataset) datasets comparing PseudoSorter with recent, single-channel spike sorting algorithms: ensemble of autoencoders (AE-Ensemble) (*31*), ROSS (*33*), and improved deep embedding clustering (IDEC) (*37*, *49*). AE-Ensemble applies multiple autoencoders to learn unsupervised feature representations before clustering, while IDEC refines deep clustering by iteratively improving cluster assignments alongside feature embeddings. ROSS is an automatic spike sorting algorithm that uses adaptive multipoint spike detection and clustering based on a mixture of skew-t distributions. In addition, we include PCA-GMM (*24*, *25*) and PCA + HDBSCAN (implemented in SpikeInterface as “Simple Sorter”) (*19*) as established traditional approaches that use PCA for feature selection combined with Gaussian mixture models (GMMs) and hierarchical density-based spatial clustering of applications with noise (HDBSCAN) (*50*) for clustering, respectively. These methods were chosen for benchmarking as they use a comparable input-to-output structure as PseudoSorter and allow direct classification of isolated spike shape recordings. Each method is evaluated five times on each dataset, and the mean accuracy is calculated to ensure robust and reliable results. For Small datasets, the median accuracy of PseudoSorter is 75.80%, which is above IDEC’s 73.82% and AE-Ensemble’s 72.97%, and substantially above PCA-GMM’s 55.88% and PCA + HDBSCAN’s 55.74%. ROSS displays the lowest median accuracy at 54.93%. Applied to the Large datasets, PseudoSorter’s median accuracy is substantially higher at 92.05%, compared to IDEC’s 85.12%, AE-Ensemble’s 71.91%, PCA-GMM’s 60.77%, and ROSS’ 56.26%. For the Large datasets, PCA + HDBSCAN demonstrates the lowest median accuracy at 50.98%.

Figure 3B explores the spike sorting performance when the complexity of the dataset is scaled up by increasing the number of neurons from 6 to 15 while keeping the size of the dataset constant (Complex datasets, 100,000 spikes per dataset). With the complexity scaled up to 7 neurons, PseudoSorter outperforms the others, achieving a median accuracy of 80.39% compared to AE-Ensemble’s 68.98%, IDEC’s 70.94%, PCA-GMM’s 53.44%, PCA + HDBSCAN’s 49.61%, and ROSS’ 46.46%. This performance gap narrows as the complexity continues to increase; however, PseudoSorter consistently outperforms the others in terms of median accuracy. For instance, with 10 neurons, PseudoSorter registers a median accuracy of 64.44%, whereas AE-Ensemble’s, IDEC’s, PCA-GMM’s, PCA + HDBSCAN’s, and ROSS’ median accuracies drop to 59.63, 51.93, 44.05, 39.09, and 38.30%, respectively. At the highest complexity level involving 15 neurons, PseudoSorter still preserves a relatively high median accuracy of 59.91%, whereas AE-Ensemble’s and IDEC’s decrease to 51.61 and 46.12%, respectively. PCA-GMM’s and PCA + HDBSCAN’s median accuracies drop, however, more strongly to 37.12 and 29.73%, respectively, while ROSS declines to 30.50%. The trend across these varied complexity levels indicates that PseudoSorter not only scales well with increased data volume, as shown Fig. 3A, but also demonstrates superior performance when faced with more intricate data structures, which is crucial for spike sorting applications.

Figure 3C shows how spike sorting performance is influenced by signal-to-noise ratio (SNR) in the neural recordings using three distinct SNR bins (4 to 6, 6 to 8, and >8, with a maximum SNR of 11.59 across all datasets). At the lowest SNR range (4 to 6), PseudoSorter achieves the highest median accuracy of 85.02%, outperforming the other methods, with IDEC at 73.60% and AE-Ensemble at 70.70%. Performance drops substantially for PCA-GMM and PCA + HDBSCAN, with accuracies of 52.33 and 49.72%, respectively, indicating limited resilience to high noise levels. In the intermediate SNR range (6 to 8), IDEC achieves a median accuracy of 79.35%, followed by PseudoSorter at 77.56%. AE-Ensemble improves to 74.38%, while the remaining methods exhibit limited gains, with PCA-GMM at 65.33%, ROSS at 57.66%, and PCA + HDBSCAN at 53.33%. These results suggest that IDEC and PseudoSorter maintain robustness in moderately noisy conditions. In the highest SNR bin (>8, up to 11.59), PseudoSorter achieves again the highest median accuracy of 86.96%, leveraging the improved signal quality. IDEC achieves a median accuracy of 82.67%, while AE-Ensemble follows at 70.14%. The other methods perform less effectively, with accuracies between 52 and 54%. These results highlight PseudoSorter’s adaptability across all SNR conditions, particularly in high-noise (low-SNR) conditions and when fully using the improved signal quality at higher SNRs. This robustness across a range of noise levels is crucial for real-world spike sorting applications, where signal quality can vary substantially.

Another important metric for the quality of a spike sorting approach is its ability to identify the correct number of signal sources, i.e., neurons. This is a critical aspect of spike sorting, as accurate identification of neuron numbers is essential for subsequent analyses. Thus, the predictive performance of each method regarding neuron numbers is investigated as the complexity of the datasets increases (Fig. 3D and fig. S1 for Small and Large datasets). Across all levels of complexity, it is observed that all methods, apart from PCA + HDBSCAN, tend to strongly underestimate the number of neurons. As dataset complexity grows, PCA + HDBSCAN, PseudoSorter, and ROSS exhibit a mild upward trend in predicted neuron numbers, indicating some adaptability to complexity. Conversely, PCA-GMM, IDEC, and the AE-Ensemble predictions remain consistently low, showing no clear increase with greater complexity. Among the methods, PCA + HDBSCAN’s median predictions are the least underestimated; however, it shows high variance in its predictions. Furthermore, although PCA + HDBSCAN’s predicted number of neurons is closest to the ground truth, its accuracy is among the lowest of the spike sorters, indicating that the identified clusters do not correspond well to the actual neurons. PseudoSorter follows PCA + HDBSCAN in terms of neuron number predictions, with IDEC and the AE-Ensemble showing the most substantial underprediction, and PCA-GMM and ROSS falling in between but displaying considerable variance. Note that in Fig. 3B, the number of neurons is provided as prior information to the spike sorting algorithm. In contrast, Fig. 3D evaluates the algorithm’s ability to estimate the number of neurons without this prior knowledge, which is a more challenging task. As a result, while classification accuracy remains stable, the deviation from the ground-truth neuron count increases as dataset complexity grows.

### PseudoSorter reveals that Tau impairs a subclass of hippocampal neurons

**Fig. 4.**
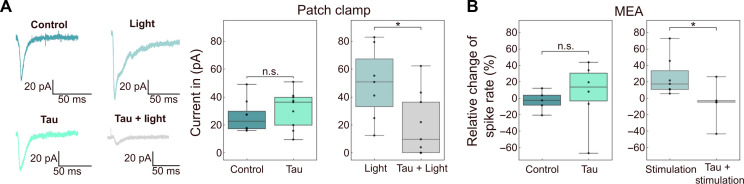
Electrophysiological experiments reveal neuronal activity-dependent effects of Tau. (**A**) Whole-cell voltage-clamp data of hippocampal neurons in the presence or absence of Tau treatment and light-stimulated activity (right), and example traces showing EPSCs recorded from neurons after the different treatment/stimulation conditions (left). *N* ≥ 7. n.s. (not significant) *P* > 0.05; *P ≤ 0.05. (**B**) Relative change of spike rate measured on MEAs following different treatments using Tau + electrical stimulation, compared to the pretreatment baseline. *N* ≥ 5. n.s. (not significant) *P* > 0.05; *P ≤ 0.05.

To first assess the effect of the addition of subneuronal concentrations of extracellular Tau to hippocampal neurons, we have performed whole-cell voltage-clamp experiments using ChR2-EYFP hippocampal neurons. ChR2-EYFP is a fusion protein combining Channelrhodopsin-2, a light-activated ion channel, with enhanced yellow fluorescent protein for optogenetic control and visualization. Excitatory neurons (CA3 hippocampus) are optogenetically stimulated and their excitatory postsynaptic currents (EPSCs) are measured in EYFP-negative neurons. Figure 4A illustrates a substantial reduction in current during Tau + optogenetic stimulation compared to the light stimulation treatment, accompanied by distinct changes in the current profile at the respective neuron. We observe that Tau-only treatment is not significantly different to the Control without Tau, while optogenetic stimulation on its own amplified the amplitude (fig. S2) and duration of the current profile. Next, and to assess whether PseudoSorter is able to shed further light on the neuronal effects of extracellular Tau, we monitor the activity of Tau-treated hippocampal neurons using MEAs in the presence and absence of electrical stimulations. Cells are treated for 2.5 hours with PBS (Control and Stimulation-only) and 1 μM Tau (Tau-only and Tau + Stimulation). During the treatment, Control and Tau-only cells are left in an incubator while Stimulation-only and Tau + Stimulation cells are electrically stimulated (see the “MEA recordings” section). We first benchmark our experimental approach without applying any specific neuronal spike classification. Our results show that Tau treatment alone, at the given low concentration, does not lead to a measurable change in spike rate compared to the Control (Fig. 4B). Meanwhile, a significant increase in spike rate is observed in cells that have been electrically stimulated (fig. S2). However, in the presence of Tau, electrical stimulation fails to induce a spike rate increase as observed in the stimulation-only condition (Fig. 4B).

**Fig. 5.**
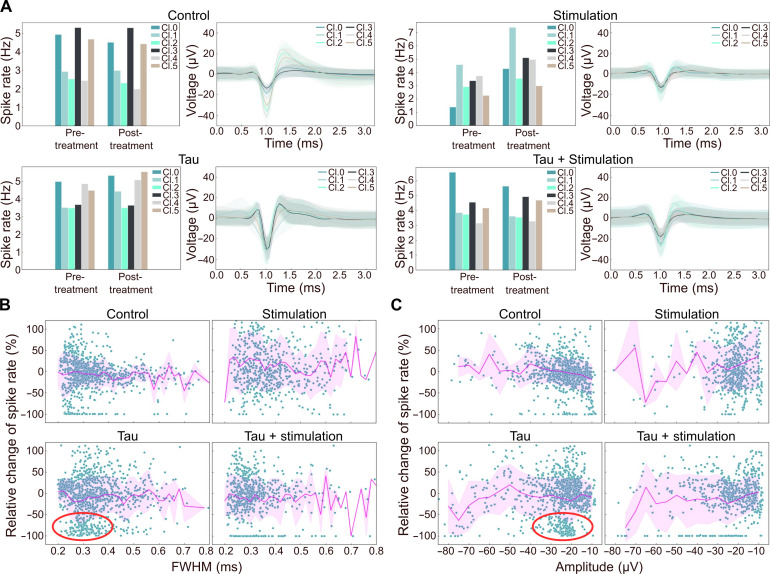
PseudoSorter analysis reveals that Tau itself already impairs a subclass of hippocampal neurons. (**A**) Demonstration of representative single electrode analysis as detected by PseudoSorter. Shown are sorted spike shapes PseudoSorter (right, called classes Cl. 0 to Cl. 5, mean ± SD in shaded area) found at single, representative electrode, and their respective firing rates before and after treatment (left) for all conditions. (**B** and **C**) Differential analysis enabled by spike sorting. Shown are relative changes in firing rate for every single spike class identified by PseudoSorter as a function of the spike classes’ respective FWHM (B) or amplitude (C). FWHM is calculated as the time difference between the two points where the waveform’s amplitude is half of its peak amplitude. Each dot represents one specific spike class found at one specific electrode with mean and SD shown as a magenta line and shaded area, respectively. Subclass of neurons with spike rate loss for Tau-only treatment are marked by red circles. Data acquired in *N* ≥ 5 recordings for each condition.

While insights from the patch-clamp experiments are limited to single neurons, PseudoSorter can provide further understanding at different scales (network versus single neurons) by analyzing spikes recorded at each electrode. Figure 5A showcases examples where PseudoSorter classified all spikes at single electrodes. Here, spike classes refer to individual neurons detected and grouped by the spike sorting algorithm. The depicted example electrodes display typical spike shapes observed during the experiment, along with their respective spike rates before and after treatment. For these example electrodes, the Tau + stimulation treatment induces only slight changes in spike rates for all classes, similar to Tau-only and the Control. As anticipated, stimulation-only shows a general increase in spike rates for all spike classes at this specific electrode.

While the analysis of individual electrodes provides insights into local effects, interpretation is challenging due to substantial variations in local environments between electrodes. We have thus explored the possibility of aggregating single neuron effects to extrapolate overall trends in Fig. 5 (B and C). Note that to confirm that the identified units correspond to single cells, we have only considered units with a refractory period violation rate (RPV rate) lower than 0.1%. The RPV rate is the number of spikes that violate the refractory period divided by the total number of spikes per class (the refractory period is set to 2 ms). Figure 5B depicts the relative change in spike rates for each single neuron class as a function of the average full width at half maximum (FWHM) of the respective spike class, revealing width-dependent effects for Tau-only treatment (highlighted by the red circle). Previous work has highlighted the suitability of FWHM to characterize and identify neuron classes (*51*, *52*). Here, a decrease in spike rate is observed for spikes with FWHM around 0.27 ms, indicating that many neurons largely or completely lost their activity in that regime. Similarly, Fig. 5C visualizes the relative change in spike rates for each single neuron class as a function of the average amplitude of the respective spike class. A slight increase in spike rate after treatment is observed for spike classes with an amplitude of approximately −20 μV compared to smaller amplitude spikes (around −10 μV) for stimulation only, Tau-only, and Tau + stimulation, which is not observed for the no-Tau Control. Consistent with Fig. 5B, many neurons of amplitudes between −30 and −20 μV are largely lost (>50% loss in spike rate) for Tau-only treatment (highlighted by the red circle).

## DISCUSSION

PseudoSorter marks an important shift in ML spike sorting methodologies by adopting the latest advances in ML. Notably, PseudoSorter represents the first application of a self-supervised framework for spike sorting (*53*). Our approach thus enhances the understanding of complex neuronal data, leading to more nuanced neuronal activity analyses. The key feature of our approach, a density-based pseudolabeling strategy, ensures adaptability and generalizability by focusing on representative spike samples. Fine-tuning with pseudolabels crucially improves signal classification accuracy, especially for complex spike samples. Furthermore, an iterative fine-tuning process, designed to explore the space of pseudolabels progressively, avoids falling into local minima due to insufficient or poor-quality pseudolabels. The improvements from each iteration are demonstrable, with clearer clustering in the latent space and enhanced overall accuracies. As a result, PseudoSorter exhibits higher classification accuracy and, thus, outperforms current ML spike sorting methods on simulated, single-channel spike recording data. This is particularly notable when scaling with increased data volumes or complexity, both of which are an important aspect given the capabilities of modern MEAs to record a vast amount of intricate neuronal signals (*54*). However, this refinement also contributes to increased computational cost. As highlighted in table S2, while PseudoSorter requires a runtime comparable to ROSS, it is computationally more expensive than AE-Ensemble and IDEC. Moreover, traditional methods such as PCA + HDBSCAN and PCA + GMM offer even faster processing times but at the cost of substantially lower classification accuracy (Fig. 3). Future improvements may consider the optimization of the fine-tuning phase, potentially through more efficient architectures, hardware acceleration, or model distillation techniques. These optimizations may enhance the usability of PseudoSorter, particularly for real-time spike sorting applications where low latency is critical.

Moreover, despite all these recent advances in the field of spike sorting, limited accuracy, especially for large number of neurons, remains (*22*). Here, the difficulty spike sorting algorithms face in accurately discerning neuron numbers in data-rich environments is explored in more detail, with our method displaying marginal superiority under these challenging conditions. Although PseudoSorter’s approach for detecting the number of neurons surpasses the presented alternatives, it is still limited, particularly when dealing with larger neuron populations. Anatomical considerations suggest that the number of neurons per electrode should be substantially higher (10×) than the generally detected up to six or seven units per electrode (*5*, *55*). The observed phenomenon can stem from several factors, such as the prevalence of silent neurons induced by electrode-related tissue damage, or limitations inherent in current spike sorting algorithms, which may struggle to distinguish the activity of numerous neurons. Our analysis indicates that, among the benchmarked algorithms, only PCA + HDBSCAN predicts more than eight source neurons; however, its predictions exhibit high variance and are accompanied by the lowest accuracy, indicating poor correspondence between the identified clusters and the actual neurons. This observation suggests that the performance of spike sorting algorithms might be a crucial bottleneck in detecting all neurons per electrode, underscoring the need for ongoing research in this area. It is imperative for future spike sorting methods to be extensively benchmarked, as satisfactory performance on small and simple datasets does not necessarily translate to more complex data obtained from biological measurements. Accordingly, it is foreseen that the datasets provided here can be used for future benchmarking studies and should undergo gradual expansion. The interpretation of spike sorting results can be challenging due to the intricate nature of neuronal data and the sophisticated algorithms used. To address this issue, an easy-to-use script is provided; however, a fundamental understanding of the algorithms remains crucial for effective optimization and troubleshooting. Moreover, reliance on simulated datasets for benchmarking algorithms may introduce biases, as these datasets may not comprehensively encompass the diversity of real neuronal data (*20*, *22*). Therefore, validation using a variety of experimental data and recognition of the limitations associated with simulated datasets are essential.

Future research in the field of self-supervised spike sorting is poised to pursue two distinct technical trajectories. First, there is a drive to enhance accuracy in handling extensive datasets, facilitated by advancements in high-density MEAs. This may involve integrating transformer models and autoregressive training methods, leveraging abundant neuronal data to achieve greater precision (*56*). Second, there is a need to integrate self-supervised learning frameworks into more compact models, enabling efficient real-time analyses of neuronal data. We anticipate that these advancements in spike sorting methodologies will not only deepen our understanding of neuronal dynamics and diseases (*57*) but also catalyze advancements in brain-computer interface development by refining the interpretation of neuronal signals (*58*).

While PseudoSorter was primarily designed for single-channel recordings, providing versatility across various study designs, we conducted preliminary tests to explore its potential for multichannel recordings (fig. S3). PseudoSorter’s architecture allows for the use of multichannel data without requiring any adjustments to the model or training procedure. Initial benchmarks against widely used multichannel spike sorters [KiloSort4 (*20*), SpyKing Circus 2 (*18*), MountainSort (*17*), and Tridescolous2 (*19*)] reveal that PseudoSorter performs well despite not incorporating targeted approaches like drift correction or template matching. However, additional testing and validation across a broader set of multichannel recordings are needed to thoroughly assess its scalability, which is beyond the scope of this study and remains an exciting direction for future research. Future work will focus on integrating targeted methods to address challenges such as drift correction and overlapping spike waveforms, particularly in dense MEA layouts.

Further, we strategically designed an experiment to assess PseudoSorter’s efficacy in extracting neuronal signaling data from intricate and challenging biological datasets. In particular, we aimed to address the molecular mechanisms underlying the treatment with subneuronal concentrations of extracellular monomeric Tau. Thus far, there has been no demonstration that low concentrations of extracellular monomeric Tau induce notable defects in neuronal signaling of hippocampal neurons.

Using PseudoSorter to track spike classes through spike sorting, we show that not all spike classes have changed their spike rate uniformly, suggesting the ability to observe differential effects for each putative neuron and revealing that monomeric Tau in the extracellular space, even at subneuronal concentrations, is able to cause specific loss of neuronal activity in a subset of hippocampal neurons. Combining network-level insights with data from single electrodes enables researchers to achieve a more comprehensive understanding at various levels (network ➔ electrode ➔ neuron), which has previously been suggested for the study of axonal dysfunction in neurodegenerative diseases (*59*). Notably, PseudoSorter proficiently extracts a subset of neurons in the Tau-only group that exhibit a decrease in spike rates for spikes with an FWHM of approximately 0.27 ms (Fig. 5, B and C), suggesting that Tau has an effect on the network activity stemming either from multiunit activity defects, or from defects in fast inhibitory (*60*) neurons, the latter of which matches observations from data from patients with AD (*61*). The bar graphs in Fig. 5A are representative of the overall data, showing spike rates of individual putative neurons detected by PseudoSorter for specific example electrodes rather than aggregated dataset-wide distributions. Therefore, statistical analysis has not been applied to these graphs. Similarly for Fig. 5 (B and C), conventional statistical tests are not well suited for evaluating these distributions, as they do not represent population-level averages but rather individual neuron-level variability. Instead, these plots aim to qualitatively illustrate the heterogeneity of neuronal responses, emphasizing that Tau-induced effects are not uniform across all neurons.

Using whole-cell voltage-clamp recordings, our data show that Tau + optogenetic stimulation leads to a reduction in EPSCs in ChR2-EYFP excitatory hippocampal neurons compared to stimulation only (Fig. 4A). Similarly, Tau treatment during heightened neuronal activity seems to mitigate the enhancing effects of the stimulation (Fig. 4B and fig. S2). In comparison to patch-clamp experiments, MEAs have not yet fully established themselves to uncover intricate neuronal signaling deficits due to the limitations in analyzing the associated complex datasets. Here, we highlight the potential of PseudoSorter to provide a more detailed analysis of the recorded data, which also comes at a much lower experimental cost in comparison to patch-clamp experiments. Nevertheless, for the specific dataset under consideration, the overall surge in activity during the stimulation condition cannot be unequivocally linked to either the width or amplitude of the corresponding spike class. Instead, an overarching observation emerges, wherein each neuron, on average, manifests heightened firing—a phenomenon mitigated by the inclusion of Tau during stimulation. This nuanced analytical approach is inferred to contribute substantially to a more comprehensive understanding and holds the potential to widen the application of MEAs to detect intricate neuronal signaling deficits related to various brain diseases. Last, an additional illustration of the potential of PseudoSorter lies in its ability to evaluate varying levels of synchronicity, as depicted in fig. S4. In this context, diverse scales of synchronicity, ranging from global (pertaining to interactions between all electrodes) to local (involving interactions among single neuron classes at each electrode), are delineated. These scales can be systematically compared to enrich our comprehension of the underlying effects (*62*, *63*).

Unlike simulated datasets where ground-truth labels allow direct benchmarking (Fig. 3), the biological dataset in Fig. 5 lacks absolute labels, making it unsuitable for reevaluating previously benchmarked methods. Instead, we use this dataset to illustrate PseudoSorter’s suitability to extract detailed insights from real-world neuronal activity. While it is possible to derive FWHM distributions without spike sorting, the key advantage of our approach is that it allows neuron-specific tracking across conditions. This enables us to assess how individual neurons respond to treatment, rather than the ensemble of all neurons.

Thus, to summarize, PseudoSorter makes important contributions to advancing the fields of ML spike sorting as well as neuroscience by offering insights into the dynamics of neural networks. This capability, achieved through leveraging PseudoSorter’s high accuracy and throughput, can be invaluable not only for studying neurodegenerative diseases and mental disorders, enhancing our understanding of their underlying mechanisms, but also for therapeutic screening of drugs against these devasting diseases.

## MATERIALS AND METHODS

### PseudoSorter

#### Python version

The presented methodology has been implemented on Python 3 using the TensorFlow (*64*) library.

#### Benchmarking datasets

The datasets introduced here are designed to serve as a comprehensive, ground-truth framework for testing and comparing emerging spike-sorting algorithms. They comprise simulated recordings of spike shapes generated using NeuroCube (*38*) in standard configuration (a single electrode, 300,000 neurons/mm^3^, 7% active neuron ratio, exponential firing rate distribution, and a 20-kHz sampling rate). In each recording, five neurons were positioned around a single electrode. A normalized distance of each neuron to the electrode was determined by random selection within a range of 0 to 1 (in increments of 0.01). Here, a normalized distance of 0 refers to neurons positioned very close to the measuring electrode (default: 10 μm from electrode edge) and 1 refers to the maximum distance at which a neuron is still considered a single unit (default: 100 μm). Firing rates were randomly chosen to range between 15 and 35 Hz. We provide the created Cube files, raw recording files in .mat file format, as well as isolated spike shape recording files, stored as Python pickle files. Here, the first column carries the ground-truth spike class for each spike (integer). The second column contains the spike time of the stimulated spike (in milliseconds). Last, columns 3 to 66 contain the respective spike shape (64 data points). The datasets are organized in size and complexity. The group of sets called Small and Large consist of 10 sets of spike recordings of five classes (source neurons) each, and include about 100,000 and 1,100,000 spike shape recordings, respectively. The Complex set group consists of 100 sets, each containing 100,000 spike shape recordings sourced from 6 to 15 neurons (10 sets each). These sets are formed by merging the 10 Small datasets, from which a specific number of source neurons and their respective recordings are randomly chosen. Subsequently, the spike shape recordings are randomly sampled to create these sets. The datasets have been designed to roughly match the noise levels of the in vitro experiments in this work. For the experiments, we detect spikes with SNRs from 5 to 30 (with most spikes having an SNR smaller than 10). As a comparison, the mean SNR (in absolute units) of the Small and Large datasets are 6.52 ± 2.23 and 8.29 ± 1.84, respectively. For the evaluation of PseudoSorter on multichannel data, we generated a set of simulated datasets with diverse drift conditions (*N* = 30 datasets, 10 datasets per drift level), aiming to capture a range of realistic experimental scenarios. The multichannel datasets were created with MEArec (*65*). Each simulation emulates a 20-min NeuroNexus-32 (NeuroNexus, USA) recording (32 channels) from five distinct neurons. The drift level has been adjusted by changing the slow_drift_velocity parameter from 0 μm/min (no drift) to 2.5 μm/min (slow drift) and 5 μm/min (high drift). For all other parameters, defaults have been chosen.

#### Source neuron prediction

In the case of all benchmarked methods, the range for identifying the optimal cluster count is limited from 2 to a maximum of 20 source neurons.

### Experiments on stimulation-dependent Tau pathology

#### Dissection of primary rat neurons

Hippocampal neurons of Sprague-Dawley rats (Charles River, UK), at 2 days postnatal (P2), were excised and gathered into 2-ml Eppendorf tubes filled with cold Dulbecco’s modified Eagle’s medium (DMEM; Sigma-Aldrich, UK) and kept chilled on ice. Following the collection of tissue, the initial cold DMEM was replaced with DMEM at room temperature, supplemented with 0.1% trypsin and 0.05% DNase (Sigma-Aldrich, UK). The tubes were subsequently placed in a CO_2_ incubator maintained at 37°C, with 5% carbon dioxide and 20% humidity, for a duration of 20 min. The cells underwent four washes with DMEM containing 0.05% DNase at room temperature and were dispersed into a single-cell suspension through gentle pipetting, first with a 1-ml and then with a 200-μl Gilson pipette tip. Following centrifugation of the cell suspension at 600 rpm for 5 min, the supernatant was discarded, and the cell pellet was softly resuspended in DMEM containing 10% FBS. The number of cells was assessed using a hemocytometer.

#### Cell culture

After autoclaving, the MEAs were cleaned with ethanol and deionized water (3× each). They were then incubated with poly-l-lysine (PLL) solution (Sigma-Aldrich, UK) overnight. On the next day, the PLL was rinsed off three times with Dulbecco’s phosphate-buffered saline and subsequently placed under an ultraviolet lamp in a sterile laminar flow cabinet for 2 hours to activate the surface. Then, 1 ml of NbActiv4 growth medium (BrainBits, USA) was introduced. The devices were placed in an incubator set at 37°C, 5% carbon dioxide, and 20% relative humidity, to warm up before plating primary P2 hippocampal neurons. A total of 180,000 primary hippocampal cells were then plated directly onto the device. Medium (200 μl) was taken out and replaced with 300 μl of warmed up NbActiv4 medium every other day to maintain the cell culture in an incubator set at 37°C, 5% carbon dioxide, and 20% relative humidity until the devices were used for experiments on days in vitro (DIV) 21.

#### Expression and purification of htau40

The human microtubule-associated protein tau (htau40) was recombinantly expressed using the pET29b vector in the *Escherichia coli* strain BL21(DE3)-CondonPlus-RIPL (Agilent, USA) after transformation. The plasmid was obtained from Addgene (Plasmid no. 16316). The protein expression was induced using IPTG (isopropyl β-d-1-thiogalactopyranoside) as previously described (*66*). Briefly, a single colony was inoculated into 5 ml of LB (Lysogeny Broth) with kanamycin (50 μg/ml) and grown overnight at 37°C. Subsequently, 1 ml of the overnight culture was transferred to 400 ml of LB media and grown at 37°C with agitation at 220 rpm until an optical density at 600 nm (OD_600_) of 0.6 was reached, following which the culture was induced with 0.5 mM IPTG and further cultivated at 18°C for overnight. The cell pellet was collected by centrifugation at 10,000*g* for 10 min, resuspended in MES buffer [20 mM MES (2-(*N*-morpholino)ethanesulfonic acid), 1 mM EGTA (ethylene glycol-bis(β-aminoethyl ether)-*N*,*N*,*N*′,*N*′-tetraacetic acid), 0.2 mM MgCl_2_ (magnesium chloride), 5 mM DTT (dithiothreitol), and 0.1 mM PMSF (phenylmethylsulfonyl fluoride), pH 6.8] supplied with cOmplete Protease Inhibitor Cocktail, and lysed using an Ultrasonic Processor XL sonicator (Heat Systems, USA) with a cycle of 5 s on and 5 s off for a total of 5 min until the suspension became less opaque. The cell lysate was supplemented with extra NaCl (sodium chloride) to a final concentration of 0.5 M and further boiled in a water bath at 90°C for 20 min before cooling on ice. Subsequently, the cell lysate was centrifuged at 30,000*g* for 20 min before the supernatant was poured out and filtered through a 0.45-μm Sartorius Minisart NML syringe filter. The soluble fraction was dialyzed overnight at 4°C in a dialysis buffer [20 mM MES, 1 mM EGTA, 1 mM MgCl_2_, 2 mM DTT, and 0.1 mM PMSF, pH 6.8, using NaOH (sodium hydroxide)] to make sure that the concentration of NaCl is less than 10 mM, before proceeding with the purification step.

To check the presence of htau40 in the soluble fraction after dialysis, the samples were combined with SDS loading buffer [20% glycerol, 100 mM tris-HCl (tris(hydroxymethyl)aminomethane-hydrochloride), 4% SDS (sodium dodecyl sulfate), and 0.2% bromophenol blue, pH 6.8] and boiled at 90°C for 10 min and loaded onto NuPAGE Novex 10% bis-tris protein gels (Thermo Fisher Scientific, UK) and run for 45 min at 180 V. The dialyzed protein underwent purification via ion exchange chromatography against a linear gradient of buffer B on a HiPrep Q FF 16/10 anion exchange column (GE Healthcare, Sweden) in buffer A. Buffer A consisted of 20 mM MES, 1 mM EGTA, 1 mM MgCl_2_, 2 mM DTT, and 0.1 mM PMSF, pH 6.8, while buffer B contained 20 mM MES, 1 mM EGTA, 1 mM MgCl_2_, 2 mM DTT, 0.1 mM PMSF, and 250 mM NaCl, pH 6.8. The elution fraction was further purified using size exclusion chromatography on a Superdex 200 16/60 column in Hepes buffer [10 mM Hepes (4-(2-hydroxyethyl)-1-piperazineethanesulfonic acid), 100 mM NaCl, and 1 mM TCEP (tris(2-carboxyethyl)phosphine), pH 7.5]. The purification process was carried out on an ÄKTA Pure (GE Healthcare, USA) and htau40 was concentrated using 10k MWCO Amicon centrifugal filtration devices (Merck KGaA, Germany). The purified proteins were flash frozen in liquid nitrogen and stored at −80°C until use. Protein concentration was determined by measuring the absorbance at 280 nm using a Nanovue spectrometer and the extinction coefficient of 7450 M^−1^ cm^−1^.

To confirm the identity of htau40, protein liquid chromatography–mass spectrometry (LC-MS) analysis was conducted on the mass spectrometer Xevo G2-S coupled with the ultraperformance liquid chromatography (UPLC) system. The column used for the LC was Acquity UPLC Protein BEHC4 with dimensions 2.1 mm by 50 mm. The gradient for the method was as follows: time 0 min, flow rate 0.2 ml/min for composition 95% A solution (water with 0.1% formic acid) and 5% B (Acetonitrile); time 1.00, 95% A and 5% B; time 5 min, 0% A and 100% B; time 6 min, 0% A and 100% B; and time 7 min, 95% A and 5% B. The data were processed by MassLynx software that controls LC-MS analysis and runs Xevo. See additional data and figures (fig. S5) for the protein sequence and purification result of htau40.

#### MEA recordings

MEA recordings were performed using a Multi Channel Systems recording setup (Multi Channel Systems, Germany). A microscope stage-top incubator (OKOLab, Italy) was placed directly onto the MEA head stage during recording sessions and was maintained at 37°C, 5% carbon dioxide, and 20% relative humidity. On DIV 21, electrophysiological recordings were obtained. MEAs used are a mix of indium tin oxide MEA devices (*67*) and PEDOT:PSS Poly(3,4-ethylenedioxythiophene) polystyrene sulfonate MEAs developed in the group (*68*). All MEAs consist of 60 electrodes that are arranged in an 8 by 8 grid (without corner electrodes). The electrodes have a diameter of 30 μm and are spaced 200 μm apart. First, a baseline recording of 30 min of spontaneous activity was acquired. Subsequently, cells were treated for 2.5 hours with one of the treatment conditions, i.e., PBS (Control), PBS (Stimulation-only), and 1 μM Tau (Tau-only and Tau + Stimulation). For Control and Tau-only, the cells were kept in an incubator (37°C, 5% carbon dioxide, and 20% relative humidity) during the treatment period. For Stimulation-only and Tau + Stimulation, cells were kept in the MEA head stage with the OKOLab setup and subjected to the stimulation protocol for the full treatment period. After treatment, another 30 min of spontaneous activity was recorded. For the stimulation (used for stimulated activity measurements as well as for treatment stimulation), we used three monophasic pulses (amplitude −700 mV) of 200-μs length and 33-ms intervals between each pulse. These stimulation pulses are then repeated every 10 s.

#### Spike detection and preprocessing of MEA recordings

Experimentally acquired raw MEA recordings were first filtered. As a common practice, bandpass filtering between 300 and 3000 Hz was applied. Subsequently, spike events were detected and isolated from the extracellular recording via thresholding. Thresholding was based on an estimate of the background noise $\sigma_{m}$

σm=median∣x∣0.6745

where *x* is the bandpass filtered signal. The threshold condition was then defined as (*13*)

$$\text{Threshold}=5\;\sigma_{m}$$

Usually, 64 sampling points were extracted for each spiking event (20 sampling points before the threshold event plus 44 sampling points after the event). The detected spike events were aligned based on the occurrence time or their minimum amplitude and further preprocessed: All spikes were min–max normalized to a scale of 0 to 1 and mapped to their gradient as it was more amenable to signal processing (*69*). Every spike recording $x_{i}\left(t\right)$ of the dataset $X=\left\{x_{1},x_{2},\ldots ,x_{n}\right\},x_{n}\in R^{d}$ was mapped to a gradient representation $\nabla x_{i}\left(t\right)$ as

$$\nabla x_{i}\left(t\right)=\frac{x_{i}\left(t+1\right)-x_{i}\left(t\right)}{∆t}$$

where t is the sampling time of each sampling point t∈(0,d−1) and ∆t is the sampling step time (50 μs for recordings obtained at a frequency of 20 kHz).

#### Patch clamp

For patch-clamp recordings, primary hippocampal cultures were prepared from P0/P1 pups from Grik4-cre mice crossbred with Ai32(RCL-ChR2(H134R)/EYFP) (the Jackson Laboratory, USA) that express ChR2-EYFP in CA3 hippocampal neurons. Recordings were carried out after 14 to 21 DIV depending on the expression levels of ChR2-EYFP (DIV14 to 21). Coverslips were incubated and stimulated applying the following conditions: Control, light stimulation, Tau (1 μM), or Tau (1 μM) + light stimulation. For the stimulation, we used three monophasic pulses of 1-ms length and 33-ms intervals between each pulse. Slightly longer pulses than the electrical stimulation in MEA experiments (1 ms compared to 200 μs, respectively) were chosen as required to evoke reliable responses. These stimulation pulses were then repeated every 10 s. Individual coverslips were transferred to an immersion-type recording chamber and superfused with artificial cerebrospinal fluid (126 mM NaCl, 3 mM KCl, 26.4 mM NaH_2_CO_3_, 1.25 mM NaH_2_PO_4_, 2 mM MgSO_4_, 2 mM CaCl_2_, and 10 mM glucose, pH 7.2, and osmolarity 270 to 290 mosmol liter^−1^). Patch pipettes were made from borosilicate glass capillaries (0.68 mm inner diameter, 1.2 mm outer diameter) (World Precision Instruments, UK) using a P-97 Flaming/Brown Micropipette Puller (Sutter Instrument, USA) with tip resistances of 4 to 7 megohms. Neurons were visualized and selected using infrared differential interference contrast microscopy using a 40× water-immersion objective. EYFP-negative neurons were identified using a U-RFL-T mercury light source (Olympus, Japan) with excitation filter 490 to 550 nm through the objective and selected for whole-cell patch-clamp recordings in voltage clamp mode. EPSCs were evoked by optogenetic stimulation (single 1-ms pulses, repeated every 20 s) of ChR2-expressing neurons using a DPL-473 laser controlled by a UGA-40 point laser system (3.5–5 mW laser intensity, Rapp OptoElectronic, Germany). For each set of recordings, the laser was adjusted using the Control condition and kept the same for all four conditions. Data were acquired using an ITC18 interface board (Instrutech, USA). At least 10 EPSC traces were averaged per cell and the amplitude was analyzed using Igor Pro software (WaveMetrics, USA).

#### Statistical analysis

GraphPad Prism 9.5.1 was used for all statistical evaluations. All datasets and comparisons have been tested for normality as well as homogeneity of variances using a Shapiro-Wilk and *F* test, respectively. Accordingly, means have been compared using two-sided Student’s *t* tests. For the comparison between the MEA datasets for the Control and Tau conditions (Fig. 4B), a Welch’s *t* test was applied.

## Supplementary Materials

### This PDF file includes:

Figs. S1 to S5 Tables S1 and S2 Supplementary Text S1 References
