# Supplementary material for: PseudoSorter: A self-supervised spike sorting approach applied to reveal Tau-induced reductions in neuronal activity
Source: Sci Adv. 2025 Mar 14;11(11):eadr4155. doi: 10.1126/sciadv.adr4155 (PMC11908484; doi:10.1126/sciadv.adr4155)
Supplement: Supplementary file 1 — Figs. S1 to S5 Tables S1 and S2 Supplementary Text S1 References [file sciadv.adr4155_sm.pdf]

Science Advances
AAAS

Supplementary Materials for

PseudoSorter: A self-supervised spike sorting approach applied to reveal Tau-induced reductions in neuronal activity

Marius Brockhoff *et al.*

Corresponding author: Gabriele S. Kaminski Schierle, gsk20@cam.ac.uk

Sci. Adv. **11**, eadr4155 (2025)
DOI: 10.1126/sciadv.adr4155

This PDF file includes:

Figs. S1 to S5
Tables S1 and S2
Text S1
References

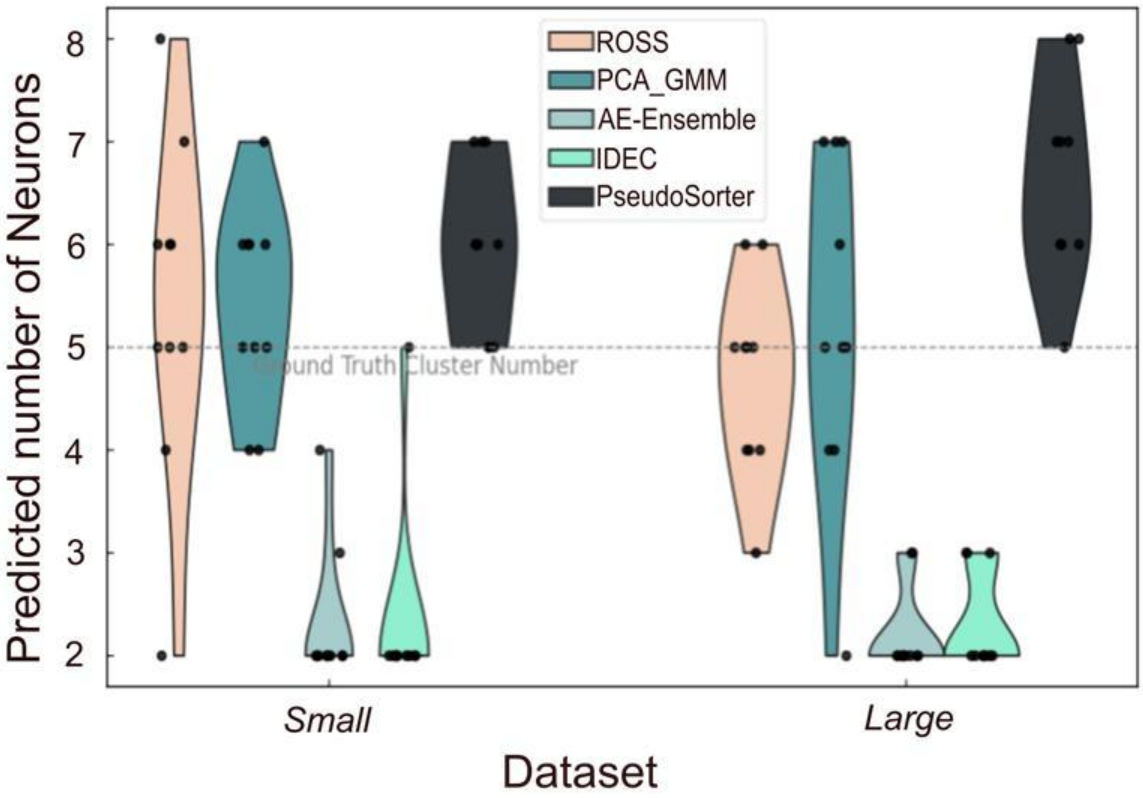

Figure S1. Violin plots showing the distribution of predicted neuron numbers for Small (5 neurons) and Large (5 neurons, increased spike samples) datasets across N=20 datasets (10 each). PseudoSorter exhibits a slight overestimation, converging around 6 neurons, while AE-Ensemble and IDEC tend to underestimate, with predictions clustering near 2 neurons. ROSS predictions display broader variability, aligning with the true neuron count or exceeding it by one for Small datasets and showing reduced variation for Large datasets. Predictions from PCA+HDBSCAN are excluded for clarity due to consistent overestimation exceeding 100 neurons for Large datasets.

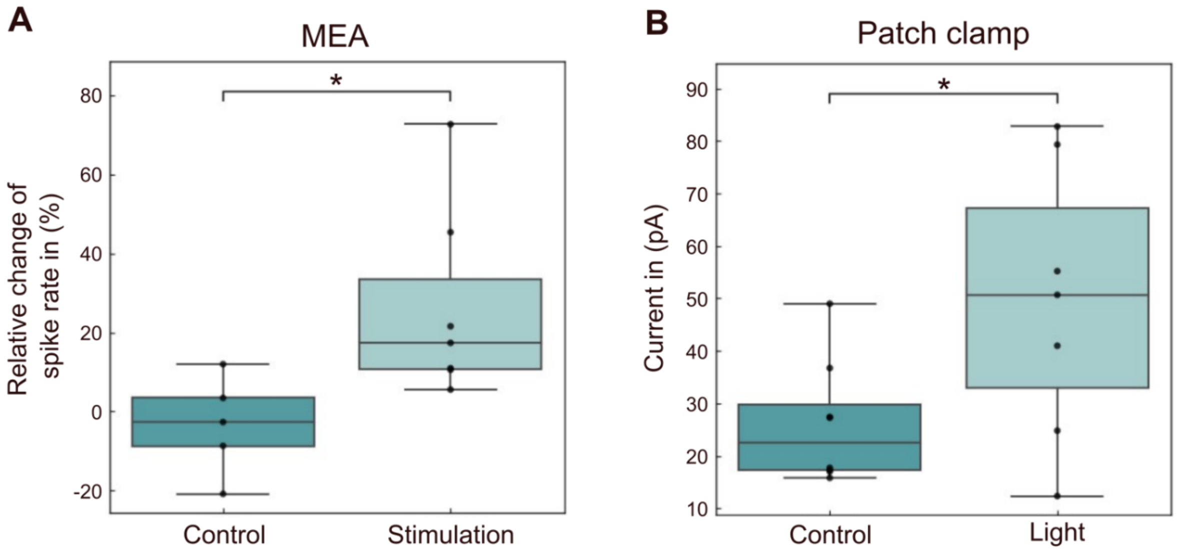

Figure S2. Stimulation increases activity and EPSCs in hippocampal neurons. (A) Relative change of spike rate measured on MEAs in the presence or absence of electrical stimulation, normalised to the pre-treatment baseline. N>=6. $^*P \leq 0.05$. **(B)** Patch clamp data of hippocampal neurons in the presence or absence of light stimulated activity. N>=7. $^*P \leq 0.05$.

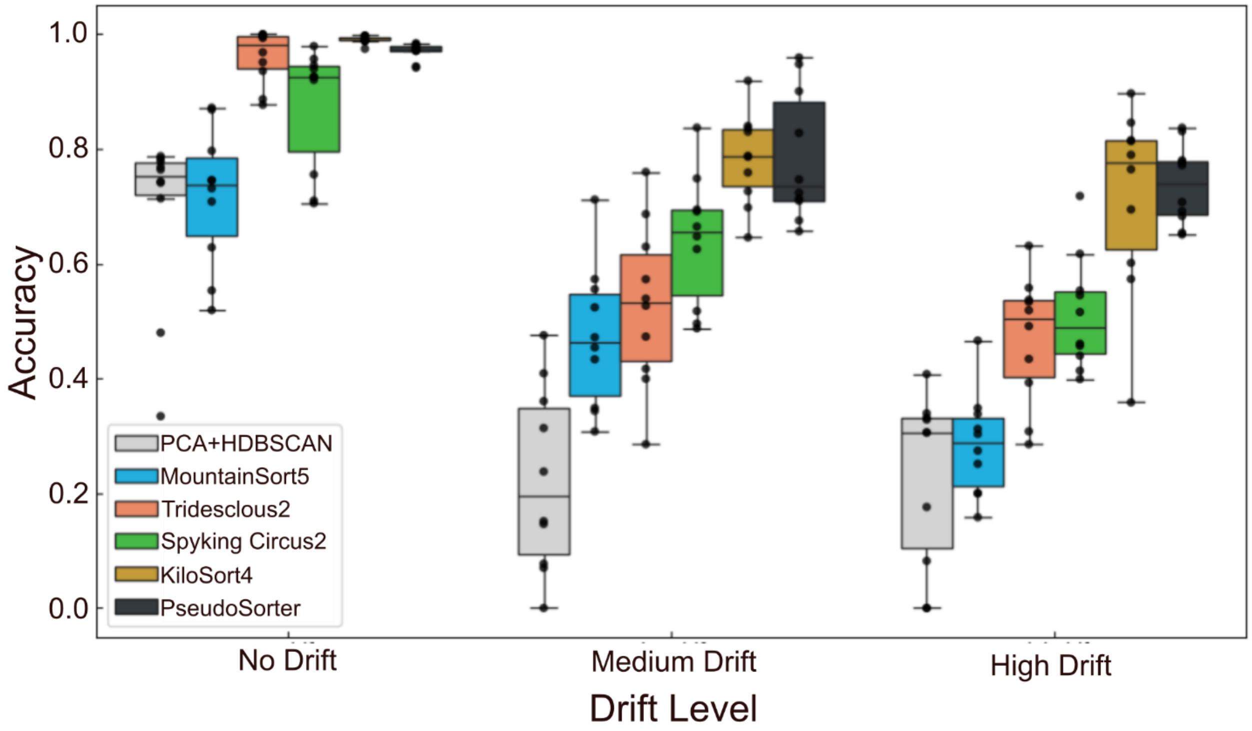

Figure S3. Multi-Channel Performance Evaluation. Benchmarking results for PseudoSorter against KiloSort4 (20), SpyKing Circus2 (18), MountainSort (17), and Tridescolous2 (19), under different drift scenarios (N=30 datasets, 10 datasets per drift level). *PseudoSorter* does not require any adjustments in its architecture to accommodate multi-channel recordings and further does not incorporate advanced features like drift correction or template matching, which are integral to the comparison methods. Results show that *PseudoSorter* achieves comparable accuracy in many cases, indicating its potential as a flexible alternative for multi-channel spike sorting.

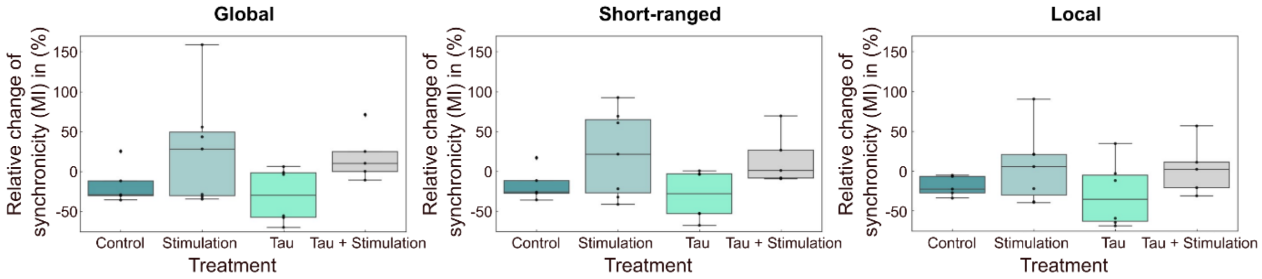

Figure S4. Spike Sorting enables multi-level synchronicity analysis. Shown are boxplots of the relative change in synchronicity (measured as mutual information (MI) (71)) after treatment compared to before treatment baseline. *Global* synchronicity measures the mutual information between all pairs of electrodes. *Short-range* synchronicity measures the mutual information between only neighbouring pairs of electrodes. *Local* synchronicity, enabled by spike sorting, describes mutual information between each class of detected neurons for every single electrode. Means of each electrode pair or across all electrodes (for local) are shown. N>=5.

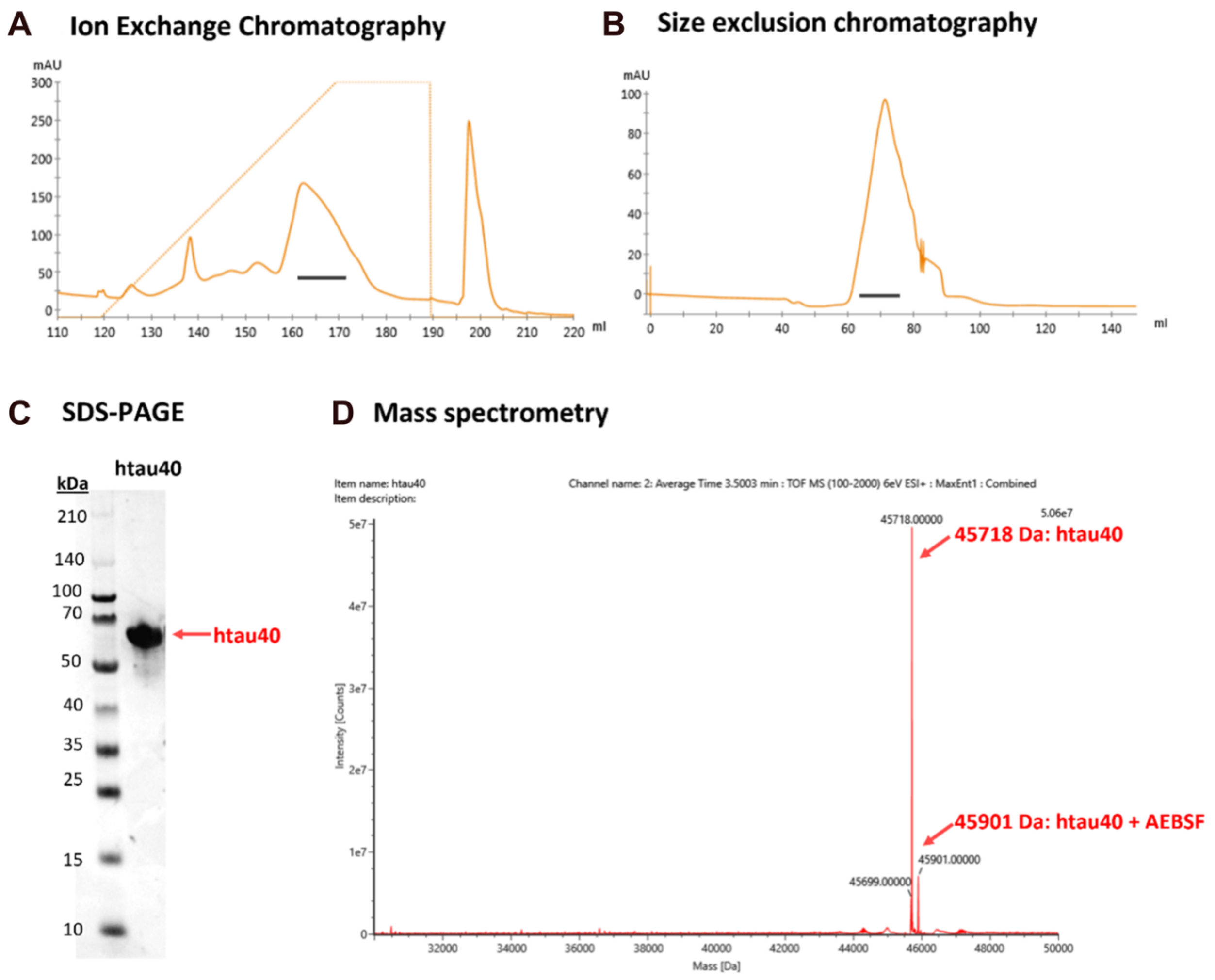

Figure S5. Sequential purification and analysis of htau40. (A) Ion-exchange chromatography using HiTrap Q HP 5 ml column with a salt gradient depicted by dashed lines for protein elution and collected protein fractions marked by black solid lines. **(B)** Further purification via size exclusion chromatography on a Superdex 200 16/60 column with collected fraction similarly denoted by solid black lines. **(C)** Analysis of the resulting protein by SDS-PAGE to verify purity. Note that its anomalous mobility (it appears to migrate as a 60 kDa protein even though its actual mass is approximate 46 kDa) is due to its intrinsically disordered nature. **(D)** Identification and characterisation of htau40 by mass spectrometry. The 45718 Da is corresponding to the full-length htau40 without N-terminal methionine (N-terminal rule), and 45901 Da indicates the free cysteine on htau40's interacted with AEBSF, an element of the cOmplete™ protease inhibitor cocktail.

Stage	Parameter	Value	Description
**Self-Supervised Pre-Training**	Pretraining Model	NNCLR	Utilises contrastive learning adapted for one-dimensional neuronal signals.
	Encoder Architecture	[63, 500, 500, 2000, 10]	Fully connected neural network with ReLU activation, 10-dimensional latent space.
	Batch Size	256	Batch size used during pretraining.
	Learning Rate	1e-3	Optimised using the Adam optimizer.
	Augmentation Type	Gaussian Noise	Maximum noise level of 0.075 to simulate spike variability.
	Temperature	0.1	Controls the sharpness of the contrastive loss.
	Queue Size	10% of dataset size	Buffer for maintaining a queue of samples for contrastive learning.
**Pseudo-Label Generation**	Sampling Strategy	Weighted and Densest Sampling	Two approaches based on local KNN density for selecting high-confidence samples.
	KNN Density Setting	0.5% of Dataset Size	Determines the local density for sampling pseudo labels.
	Clustering Method	K-means++	Clustering algorithm for pseudo-label assignment.
	Cluster Count Estimation	Elbow Method on 50% of Dataset	Estimates the number of clusters (neurons) based on the elbow method.
	Sampling Fraction	Variable	Iteratively increased to find the optimal pseudo-label ratio.
**Fine-Tuning**	Batch Size	128	Smaller batch size for iterative fine-tuning.
	Pseudo-Label Ratios	0.01, 0.05, 0.10, 0.15, 0.20, 0.30, 0.40	Ratios of pseudo-labeled samples used in successive fine-tuning iterations.
	Augmentation Type	Gaussian Noise	Maximum noise level of 0.1 to enhance robustness during classification.

Table S1: Overview of key hyperparameters and training configurations for *PseudoSorter* algorithm.

Spike Sorter	Runtime (in seconds)
PCA+HDBSCAN	100
AE-Ensemble	200
PCA + GMM	370
IDEC	600
ROSS	1950
PseudoSorter	1950

Table S2: Runtime Comparison. Overview of the run times for all tested spike sorters. Run times have been measured for sorting of 100.000 single-channel spikes on a NVIDIA A-100-SXM-80GB GPU.

Text S1: Protein Sequence htau40 (the first methionine is cleavaged due to N-dragon rule)

MAEPRQEFEVMEDHAGTYGLGDRKDQGGYTMHQDQEGDTDAGLKESPLQTPTEDGSE
EPGSETSDAKSTPTAEDVTAPLVDEGAPGKQAAAQPHTEIPEGTTAEEAGIGDTPSLEDE
AAGHVTQARMVSKSKDGTGSDDKKAKGADGKTKIATPRGAAPPGQKGQANATRIPAK
TPPAPKTPPSSGEPPKSGDRSGYSSPGSPGTPGSRSRTPSLPTPPTREPKKVAVVRTPPKSP
SSAKSRLQTAPVPMPDLKNVKSKIGSTENLKHQPGGGKVQIINKKLDLSNVQSKCGSKD
NIKHVPGGGSVQIVYKPVDLSKVTSKCGSLGNIHHKPGGGQVEVKSEKLDFKDRVQSKI
GSLDNITHVPGGGNKKIETHKLTFRENAKAKTDHGAEIVYKSPVVSGDTSPRHLSNVSST
GSIDMVDSPQLATLADEVSASLAKQGL

REFERENCES AND NOTES

1. M. E. J. Obien, K. Deligkaris, T. Bullmann, D. J. Bakkum, U. Frey, Revealing neuronal function through microelectrode array recordings. *Front. Neurosci.* **8**, 423 (2015).

2. G. Buzsáki, C. A. Anastassiou, C. Koch The origin of extracellular fields and currents—EEG, ECoG, LFP and spikes. *Nat. Rev. Neurosci.* **13**, 407–420 (2012).

3. M. E. Spira, A. Hai, Multi-electrode array technologies for neuroscience and cardiology. *Nano Enabled Med. Appl.*, 567–602 (2020).

4. E. N. Brown, R. E. Kass, P. P. Mitra Multiple neural spike train data analysis: State-of-the-art and future challenges. *Nat. Neurosci.* **7**, 456–461 (2004).

5. R. Q. Quiroga, Spike sorting. *Curr. Biol.* **22**, R45–R46 (2012).

6. C. Anastassiou, S. Panzeri, Biophysics of extracellular spikes, in *Principles of Neural Coding* (CRC Press, 2013), pp. 15–36.

7. H. G. Rey, C. Pedreira, R. Quian Quiroga Past, present and future of spike sorting techniques. *Brain Res. Bull.* **119**, 106–117 (2015).

8. D. Carlson, L. Carin, Continuing progress of spike sorting in the era of big data. *Curr. Opin. Neurobiol.* **55**:90–96 (2019).

9. P. Barthó, H. Hirase, L. Monconduit, M. Zugaro, K. D. Harris, G. Buzsáki Characterization of neocortical principal cells and interneurons by network interactions and extracellular features. *J. Neurophysiol.* **92**, 600–608 (2004).

10. M. C. Quirk, D. L. Sosulski, C. E. Feierstein, N. Uchida, Z. F. Mainen, A defined network of fast-spiking interneurons in orbitofrontal cortex: Responses to behavioral contingencies and ketamine administration. *Front. Syst. Neurosci.* **3**, 13 (2009).

11. A. R. Neumann, R. Raedt, H. W. Steenland, M. Sprengers, K. Bzymek, Z. Navratilova, L. Mesina, J. Xie, V. Lapointe, F. Kloosterman, K. Vonck, P. A. J. M. Boon, I. Soltesz, B. L.

McNaughton, A. Luczak Involvement of fast-spiking cells in ictal sequences during spontaneous seizures in rats with chronic temporal lobe epilepsy. *Brain* **140**, 2355–2369 (2017).

12. K. D. Harris, D. A. Henze, J. Csicsvari, H. Hirase, G. Buzsáki Accuracy of tetrode spike separation as determined by simultaneous intracellular and extracellular measurements. *J. Neurophysiol.* **84**, 401–414 (2000).

13. R. Q. Quiroga, Z. Nadasdy, Y. Ben-Shaul Unsupervised spike detection and sorting with wavelets and superparamagnetic clustering. *Neural Comput.* **16**, 1661–1687 (2004).

14. U. Rutishauser, E. M. Schuman, A. N. Mamelak Online detection and sorting of extracellularly recorded action potentials in human medial temporal lobe recordings, in vivo. *J. Neurosci. Methods* **154**, 204–224 (2006).

15. S. N. Kadir, D. F. M. Goodman, K. D. Harris High-dimensional cluster analysis with the masked EM algorithm. *Neural Comput.* **26**, 2379–2394 (2014).

16. C. Rossant, S. N. Kadir, D. F. M. Goodman, J. Schulman, M. L. D. Hunter, A. B. Saleem, A. Grosmark, M. Belluscio, G. H. Denfield, A. S. Ecker, A. S. Tolias, S. Solomon, G. Buzsáki, M. Carandini, K. D. Harris Spike sorting for large, dense electrode arrays. *Nat. Neurosci.* **19**, 634–641 (2016).

17. J. E. Chung, J. F. Magland, A. H. Barnett, V. M. Tolosa, A. C. Tooker, K. Y. Lee, K. G. Shah, S. H. Felix, L. M. Frank, L. F. Greengard A fully automated approach to spike sorting. *Neuron* **95**, 1381–1394.e6 (2017).

18. P. Yger, G. L.B. Spampinato, E. Esposito, B. Lefebvre, S. Deny, C. Gardella, M. Stimberg, F. Jetter, G. Zeck, S. Picaud, J. Duebel, O. Marre A spike sorting toolbox for up to thousands of electrodes validated with ground truth recordings in vitro and in vivo. *eLife* **7**, e34518 (2018).

19. A. P. Buccino, C. L. Hurwitz, S. Garcia, J. Magland, J. H. Siegle, R. Hurwitz, M. H. Hennig, SpikeInterface, a unified framework for spike sorting. *eLife* **9**, e61834 (2020).

20. M. Pachitariu, S. Sridhar, J. Pennington, C. Stringer Spike sorting with Kilosort4. *Nat. Methods* **21**, 914–921 (2024).

21. S. Gibson, J. W. Judy, D. Markovic Spike sorting: The first step in decoding the brain: The first step in decoding the brain. *IEEE Signal Process. Mag.* **29**, 124–143 (2012).

22. A. P. Buccino, S. Garcia, P. Yger Spike sorting: New trends and challenges of the era of high-density probes. *Prog. Biomed. Eng.* **4**, 022005 (2022).

23. S. Shoham, D. H. O'Connor, R. Segev How silent is the brain: Is there a "dark matter" problem in neuroscience? *J. Comp. Physiol. A Neuroethol. Sens. Neural Behav. Physiol.* **192**, 777–784 (2006).

24. M. S. Lewicki, A review of methods for spike sorting: The detection and classification of neural action potentials. *Netw. Comput. Neural Syst.* **9**, R53–R78 (1998).

25. S. Shoham, M. R. Fellows, R. A. Normann Robust, automatic spike sorting using mixtures of multivariate t-distributions. *J. Neurosci. Methods* **127**, 111–122 (2003).

26. T. Wu, W. Zhao, E. Keefer, Z. Yang Deep compressive autoencoder for action potential compression in large-scale neural recording. *J. Neural Eng.* **15**, 066019 (2018).

27. T. Wu, A. Rátkai, K. Schlett, L. Grand, Z. Yang, Learning to sort: Few-shot spike sorting with adversarial representation learning, in *2019 41st Annual International Conference of the IEEE Engineering in Medicine and Biology Society (EMBC) [Internet]* (IEEE, 2019), pp. 713–716.

28. J. Lee, C. Mitelut, H. Shokri, I. Kinsella, N. Dethe, S. Wu, K. Li, E. B. Reyes, D. Turcu, E. Batty, Y. J. Kim, N. Brackbill, A. Kling, G. Goetz, E.J. Chichilnisky, D. Carlson, L. Paninski, YASS: Yet another spike sorter applied to large-scale multi-electrode array recordings in primate retina. bioRxiv 997924 [Preprint] (2020). http://biorxiv.org/lookup/doi/10.1101/2020.03.18.997924.

29. Z. Li, Y. Wang, N. Zhang, X. Li An accurate and robust method for spike sorting based on convolutional neural networks. *Brain Sci.* **10**, 835 (2020).

30. M. Rácz, C. Liber, E. Németh, R. Fiáth, J. Rokai, I. Harmati, I. Ulbert, G. Márton Spike detection and sorting with deep learning. *J. Neural Eng.* **17**, 016038 (2020).

31. J. Eom, I. Y. Park, S. Kim, H. Jang, S. Park, Y. Huh, D. Hwang Deep-learned spike representations and sorting via an ensemble of auto-encoders. *Neural Netw.* **134**, 131–142 (2021).

32. J. Rokai, M. Rácz, R. Fiáth, I. Ulbert, G. Márton, ELVISort: Encoding latent variables for instant sorting, an artificial intelligence-based end-to-end solution. *J. Neural Eng.* **18**, 10.1088/1741-2552/abf521 (2021).

33. R. Toosi, M. A. Akhaee, M.R. A. Dehaqani An automatic spike sorting algorithm based on adaptive spike detection and a mixture of skew-t distributions. *Sci. Rep.* **11**, 13925 (2021).

34. D. Valencia, A. Alimohammad, Neural spike sorting using binarized neural networks. *IEEE Trans. Neural Syst. Rehabil. Eng.* **29**, 206–214 (2021).

35. J. Wouters, F. Kloosterman, A. Bertrand A data-driven spike sorting feature map for resolving spike overlap in the feature space. *J. Neural Eng.* **18**, 10.1088/1741-2552/ac0f4a (2021).

36. M. Saif-ur-Rehman, O. Ali, C. Klaes, I. Iossifidis, Adaptive SpikeDeep-Classifier: Self-organizing and self-supervised machine learning algorithm for online spike sorting. arXiv:2304.01355 [q-bio.NC] (2023).

37. M. Lu, E. Hui, M. Brockhoff, J. Träuble, A. Fernandez-Villegas, O. J. Burton, J. Lamb, E. Ward, P. J. Woodhams, W. Tadbier, N. F. Läubli, S. Hofmann, C. F. Kaminski, A. Lombardo, G. S. K. Schierle, Graphene microelectrode arrays, 4D structured illumination microscopy, and a machine learning spike sorting algorithm permit the analysis of ultrastructural neuronal changes during neuronal signaling in a model of Niemann-Pick disease type C. *Adv. Sci.* **28**, 2402967 (2024).

38. L. A. Camuñas-Mesa, R. Q. Quiroga, A detailed and fast model of extracellular recordings. *Neural Comput.* **25**, 1191–1212 (2013).

39. E. M. Mandelkow, E. Mandelkow, Tau in Alzheimer's disease. *Trends Cell Biol.* **8**, 425–427 (1998).

40. C. H. Michel, S. Kumar, D. Pinotsi, A. Tunnacliffe, P. St. George-Hyslop, E. Mandelkow, E.M. Mandelkow, C. F. Kaminski, G. S. Kaminski Schierle Extracellular monomeric tau protein is sufficient to initiate the spread of tau protein pathology. *J. Biol. Chem.* **289**, 956–967 (2014).

41. N. Kfoury, B. B. Holmes, H. Jiang, D. M. Holtzman, M. I. Diamond Trans-cellular propagation of tau aggregation by fibrillar species. *J. Biol. Chem.* **287**, 19440–19451 (2012).

42. J. W. Wu, S. A. Hussaini, I. M. Bastille, G. A. Rodriguez, A. Mrejeru, K. Rilett, D. W. Sanders, C. Cook, H. Fu, R. A. C. M. Boonen, M. Herman, E. Nahmani, S. Emrani, Y. H. Figueroa, M. I. Diamond, C. L. Clelland, S. Wray, K. E. Duff Neuronal activity enhances tau propagation and tau pathology in vivo. *Nat. Neurosci.* **19**, 1085–1092 (2016).

43. K. A. Butner, M. W. Kirschner, Tau protein binds to microtubules through a flexible array of distributed weak sites. *J. Cell Biol.* **115**, 717–730 (1991).

44. S Khatoon, I. Grundke-Iqbal, K. Iqbal Brain levels of microtubule-associated protein τ are elevated in Alzheimer's disease: A radioimmuno-slot-blot assay for nanograms of the protein. *J. Neurochem.* **59**, 750–753 (1992).

45. D. Dwibedi, Y. Aytar, J. Tompson, P. Sermanet, A. Zisserman, With a little help from my friends: Nearest-neighbor contrastive learning of visual representations. *Proc IEEECVF Int Conf Comput Vis ICCV*. 9588–9597 (2021).

46. D. Arthur, S. Vassilvitskii, k-means++: The advantages of careful seeding, in *Proceedings of the Eighteenth Annual ACM-SIAM Symposium on Discrete Algorithms* (2007), pp. 1027–1035.

47. R. L. Thorndike, Who belongs in the family? *Psychometrika* **18**, 267–276 (1953).

48. M. A. Syakur, B. K. Khotimah, E. M. S. Rochman, B. D. Satoto Integration K-means clustering method and elbow method for identification of the best customer profile cluster. *IOP Conf. Ser. Mater. Sci. Eng.* **336**, 012017 (2018).

49. X. Guo, L. Gao, X. Liu, J. Yin, Improved deep embedded clustering with local structure preservation, in *Proceedings of the Twenty-Sixth International Joint Conference on Artificial Intelligence* (International Joint Conferences on Artificial Intelligence Organization, 2017), pp. 1753–1759.

50. R. J. G. B. Campello, D. Moulavi, J. Sander, Density-based clustering based on hierarchical density estimates, in *Advances in Knowledge Discovery and Data Mining*, J. Pei, V. S. Tseng, L. Cao, H. Motoda, G. Xu G, Eds. (Springer Berlin Heidelberg, 2013), pp. 160–172, vol. 7819 of *Lecture Notes in Computer Science*.

51. F. Kloosterman, S. P. Layton, Z. Chen, M. A. Wilson, Bayesian decoding using unsorted spikes in the rat hippocampus. *J. Neurophysiol.* **111**, 217–227 (2014).

52. A. Luczak, N. S. Narayanan, Spectral representation—Analyzing single-unit activity in extracellularly recorded neuronal data without spike sorting. *J. Neurosci. Methods* **144**, 53–61 (2005).

53. R. Krishnan, P. Rajpurkar, E. J. Topol, Self-supervised learning in medicine and healthcare. *Nat. Biomed. Eng.* **6**, 1346–1352 (2022).

54. N. A. Steinmetz, C. Aydin, A. Lebedeva, M. Okun, M. Pachitariu, M. Bauza, M. Beau, J. Bhagat, C. Böhm, M. Broux, S. Chen, J. Colonell, R. J. Gardner, B. Karsh, F. Kloosterman, D. Kostadinov, C. Mora-Lopez, J. O'Callaghan, J. Park, J. Putzeys, B. Sauerbrei, R. J. J. van Daal, A. Z. Vollan, S. Wang, M. Welkenhuysen, Z. Ye, J. T. Dudman, B. Dutta, A. W. Hantman, K. D. Harris, A. K. Lee, E. I. Moser, J. O'Keefe, A. Renart, K. Svoboda, M. Häusser, S. Haesler, M. Carandini, T. D. Harris, Neuropixels 2.0: A miniaturized high-density probe for stable, long-term brain recordings. *Science* **372**, eabf4588 (2021).

55. D. A. Henze, Z. Borhegyi, J. Csicsvari, A. Mamiya, K. D. Harris, G. Buzsáki Intracellular features predicted by extracellular recordings in the hippocampus in vivo. *J. Neurophysiol.* **84**, 390–400 (2000).

56. S. Zhang, R. Fan, Y. Liu, S. Chen, Q. Liu, W. Zeng, Applications of transformer-based language models in bioinformatics: A survey. *Bioinform. Adv.* **3**, vbad001 (2023).

57. F. Franke, D. Jäckel, J. Dragas, J. Müller, M. Radivojevic, D. Bakkum, A. Hierlemann, High-density microelectrode array recordings and real-time spike sorting for closed-loop experiments: An emerging technology to study neural plasticity. *Front. Neural Circuits* **6**, 105 (2012).

58. S. Todorova, P. Sadtler, A. Batista, S. Chase, V. Ventura To sort or not to sort: The impact of spike-sorting on neural decoding performance. *J. Neural Eng.* **11**, 056005 (2014).

59. X. Yuan, M. Schröter, M. E. J. Obien, M. Fiscella, W. Gong, T. Kikuchi, A. Odawara, S. Noji, I. Suzuki, J. Takahashi, A. Hierlemann, U. Frey Versatile live-cell activity analysis platform for characterization of neuronal dynamics at single-cell and network level. *Nat. Commun.* **11**, 4854 (2020).

60. H.-A. Tseng, X. Han, Distinct spiking patterns of excitatory and inhibitory neurons and LFP oscillations in prefrontal cortex during sensory discrimination. *Front. Physiol.* **12**, 618307 (2021).

61. J. J. Palop, L. Mucke, Network abnormalities and interneuron dysfunction in Alzheimer disease. *Nat. Rev. Neurosci.* **17**, 777–792 (2016).

62. W. Singer, Neuronal synchrony: A versatile code for the definition of relations? *Neuron* **24**, 49–65 (1999).

63. P. J. Uhlhaas, W. Singer, Abnormal neural oscillations and synchrony in schizophrenia. *Nat. Rev. Neurosci.* **11**, 100–113 (2010).

64. TensorFlow Developers. TensorFlow [Internet]. Zenodo; 2023 [cited 2023 Nov 29]. https://zenodo.org/doi/10.5281/zenodo.4724125.

65. A. P. Buccino, G. T. Einevoll, MEArec: A fast and customizable testbench simulator for ground-truth extracellular spiking activity. *Neuroinformatics* **19**, 185–204 (2021).

66. S. Barghorn, J. Biernat, E. Mandelkow, Purification of recombinant tau protein and preparation of alzheimer-paired helical filaments in vitro, in *Amyloid Proteins* (Humana Press, 2004), pp. 35–52.

67. Multi Channel Systems MCS GmbH. Datasheet 60PedotMEA200-30iR-Au. Multi Channel Systems MCS GmbH (2019).

68. S. Middya, V. F. Curto, A. Fernández-Villegas, M. Robbins, J. Gurke, E. J. M. Moonen, G. S. Kaminski Schierle, G. G. Malliaras Microelectrode arrays for simultaneous electrophysiology and advanced optical microscopy. *Adv. Sci.* **8**, 2004434 (2021).

69. J. H. Manton, D. Applebaum, S. Ikeda, N. le Bihan Introduction to the issue on differential geometry in signal processing. *IEEE J. Sel. Top. Signal Process.* **7**, 573–575 (2013).

70. L. van der Maaten, G. Hinton, Visualizing data using t-SNE. *J. Mach. Learn Res.* **9**, 2579–2605 (2008).

71. S. Gelfman, Q. Wang, Y.-F. Lu, D. Hall, C. D. Bostick, R. Dhindsa, M. Halvorsen, K Melodi Mc Sweeney, E. Cotterill, T. Edinburgh, M. A. Beaumont, W. N. Frankel, S. Petrovski, A. S. Allen, M. J. Boland, D. B. Goldstein, S. J. Eglen, meaRtools: An R package for the analysis of neuronal networks recorded on microelectrode arrays. *PLOS Comput. Biol.* **14**, e1006506 (2018).
